# Acute hyperglycaemia leads to altered frontal lobe brain activity and reduced working memory in type 2 diabetes

**DOI:** 10.1371/journal.pone.0247753

**Published:** 2021-03-19

**Authors:** Anna Backeström, Konstantin Papadopoulos, Sture Eriksson, Tommy Olsson, Micael Andersson, Kaj Blennow, Henrik Zetterberg, Lars Nyberg, Olov Rolandsson

**Affiliations:** 1 Department of Public Health and Clinical Medicine, Family Medicine, Umeå University, Umeå, Sweden; 2 Department of Public Health and Clinical Medicine, Nutritional Research, Umeå University, Umeå, Sweden; 3 Department of Public Health and Clinical Medicine, Medicine, Umeå University, Umeå, Sweden; 4 Department of Integrative Medical Biology, Umeå University, Umeå, Sweden; 5 Umeå Center for Functional Brain Imaging, Umeå University, Umeå, Sweden; 6 Department of Psychiatry and Neurochemistry, The Sahlgrenska Academy at the University of Gothenburg, Mölndal, Sweden; 7 Clinical Neurochemistry Laboratory, Sahlgrenska University Hospital, Mölndal, Sweden; 8 Department of Neurodegenerative Disease, UCL Institute of Neurology, Queen Square, London, United Kingdom; 9 UK Dementia Research Institute at UCL, London, United Kingdom; 10 Department of Radiation Sciences, Umeå University, Umeå, Sweden; Nathan S Kline Institute, UNITED STATES

## Abstract

How acute hyperglycaemia affects memory functions and functional brain responses in individuals with and without type 2 diabetes is unclear. Our aim was to study the association between acute hyperglycaemia and working, semantic, and episodic memory in participants with type 2 diabetes compared to a sex- and age-matched control group. We also assessed the effect of hyperglycaemia on working memory–related brain activity. A total of 36 participants with type 2 diabetes and 34 controls (mean age, 66 years) underwent hyperglycaemic clamp or placebo clamp in a blinded and randomised order. Working, episodic, and semantic memory were tested. Overall, the control group had higher working memory (mean z-score 33.15 ± 0.45) than the group with type 2 diabetes (mean z-score 31.8 ± 0.44, p = 0.042) considering both the placebo and hyperglycaemic clamps. Acute hyperglycaemia did not influence episodic, semantic, or working memory performance in either group. Twenty-two of the participants (10 cases, 12 controls, mean age 69 years) were randomly invited to undergo the same clamp procedures to challenge working memory, using 1-, 2-, and 3-back, while monitoring brain activity by blood oxygen level–dependent functional magnetic resonance imaging (fMRI). The participants with type 2 diabetes had reduced working memory during the 1- and 2-back tests. fMRI during placebo clamp revealed increased BOLD signal in the left lateral frontal cortex and the anterior cingulate cortex as a function of working memory load in both groups (3>2>1). During hyperglycaemia, controls showed a similar load-dependent fMRI response, whereas the type 2 diabetes group showed decreased BOLD response from 2- to 3-back. These results suggest that impaired glucose metabolism in the brain affects working memory, possibly by reducing activity in important frontal brain areas in persons with type 2 diabetes.

## Introduction

With an aging population, Western societies will likely experience an increased prevalence of type 2 diabetes [1], which will also increase the burden of associated complications, such as cardiovascular disease, retinopathy, nephropathy, and neuropathy. Over the last two decades, cognitive dysfunction has also emerged as a complication of type 2 diabetes [2,3], and studies have shown an association between type 2 diabetes and major neurocognitive disorder [2,4]. A recent review [5] found that individuals with type 2 diabetes have a 50% increased risk of major neurocognitive disorder compared to individuals without type 2 diabetes.

Type 2 diabetes is characterised by obesity, leading to insulin resistance and beta-cell failure, which give rise to increased blood concentrations of glucose [6]. The perturbation in glucose regulation seems to influence different memory functions differently. We previously studied the effect of fasting glucose, 2-hour glucose, and insulin resistance in a non-diabetic population in which episodic, semantic, and working memory were assessed [7]. We found that fasting glucose, but not 2-hour glucose or insulin resistance, was associated with episodic memory but found no association with semantic or working memory. Fasting glucose has been speculated to affect the hippocampus, the area in the brain most important to episodic memory. Our hypothesis was based on the assumption that the hippocampus is a very sensitive area for metabolic changes in the brain [8]. The effect of glucose on different memory functions could be divergent at different stages of poor glucose regulation. We have reported that people with long-standing type 1 diabetes and no other diabetes-related complications have poorer working memory than those without diabetes [9]. The finding may be explained by hyperglycaemia or glucose variability that is associated with structural abnormalities in the prefrontal cortex [10], a brain region important to working memory. Our studies implied that episodic or working memory, but not semantic memory, is afflicted at different stages of poor glucose regulation. Sematic memory seems to be relatively spared during the course towards dementia [11] and, therefore, not as sensitive to metabolic perturbations. However, most of our understating of the association between glucose regulation and memory function is based on observational studies, but the mechanism underlying the link between memory impairment and changes in glucose control remains largely unknown.

Recent research suggests that long-term increases and fluctuations in glucose concentrations may damage the cerebrovasculature of people with diabetes [12], leading to cognitive dysfunction. Experimental studies investigating the acute effect of hyperglycaemia on cognitive function are, by their nature, limited. Investigators have used various methods to induce hyperglycaemia in different populations over varying time frames, variations that may have contributed to their divergent results [13–15]. Accordingly, a standardised hyperglycaemic clamp should be the preferred method in a testing situation. Moreover, the effect of hyperglycaemia on the brain, as assessed by functional magnetic resonance imaging (fMRI), in correlation with working memory has scarcely been studied in people with type 2 diabetes.

A previous study using a hyperinsulinaemic euglycaemic clamp identified working memory impairment in participants with type 2 diabetes [16]. Studies on the n-back working memory task using functional neuroimaging techniques found that cognitively low-performing participants without type 2 diabetes exhibit capacity-constrained activity. In this group, the blood oxygen level–dependent (BOLD) signal response in the prefrontal cortex (PFC) did not increase linearly with increasing memory load, but levelled off between 2- and 3-back loads [17]. More recent studies have indicated similar brain patterns. A study by Huang et al. [18] that included 18 participants with newly onset type 2 diabetes and 18 matched subjects with normal blood glucose levels, were tested using the Montreal cognitive assessment scale test, the Wechsler Memory Scale Chinese-revised test, and scanned using BOLD-fMRI while performing the n-back task. They found that fMRI scan identified a neural network consisting of bilateral dorsolateral prefrontal cortex (DLPFC), bilateral premotor area (PreMA), bilateral parietal lobe (PA), and anterior cingulate cortex (ACC)/supplementary motor area (SMA) that was activated during the n-back task, with right hemisphere dominance. However, only the right PA and ACC/SMA showed a load effect via quantitative analysis in the diabetes group; the activation intensity of most working memory-related brain areas for the diabetes group were lower than for the control group under three memory loads. Furthermore, they found that the activation intensity of some cognition-related areas, including the right insular lobe, left caudate nucleus, and bilateral hippocampus/parahippocampal gyrus were lower than the control group under the memory loads.

In a small study including 12 healthy participants Zanchi et al. [19] reported that during working memory processing, glucose intake decreased activation in the anterior cingulate cortex (ACC) relative to placebo, while fructose decreased activation in the ACC and sensory cortex relative to placebo and glucose. During response inhibition, glucose and fructose decreased activation in the ACC, insula and visual cortex relative to placebo. In a very recent study by Omladic et al. [20], who studied the impact of acute hyperglycaemia, assessed working memory during normal blood glucose and during hyperglycaemia induced by a hyperglycaemic clamp. Their population was 20 adolescents (mean age 15 years) with type 1 diabetes and 20 healthy controls whose spatial working memory was tested, and brain activity assessed by fMRI. Hyperglycaemia reduced the spatial working memory in participants with type 1 diabetes, whereas the control group had improved working memory during hyperglycaemia. In addition, brain activity significantly decreased in the type 1 diabetes group but slightly increased in the controls [20].

We found only one study of brain activation analysed by fMRI and working memory that used the oral glucose tolerance test to evaluate people with type 2 diabetes and controls [21]. Both groups performed equally well on the tests, but the brain activation patterns differed. Participants with type 2 diabetes exhibited hyperactivation in the right dorsolateral PFC, left middle/inferior frontal gyrus, and left parietal cortex during the more demanding test conditions, whereas control participants did not.

We hypothesised that individuals with type 2 diabetes would have poorer memory function than controls, and that acute hyperglycaemia would further impair memory function. Based on previous non-hyperglycaemic studies of healthy adults [22] and those with schizophrenia [23,24] or dementia, we also hypothesised that we would detect a decrease in the PFC area in the brain in participants with type 2 diabetes when working memory demands were heavily taxed or exceeded during hyperglycaemia. We also predicted that we would identify a decreased BOLD signal compared to controls during functional neuroimaging. Furthermore, we explored the possibility that a difference in responses between 1- to 2-back and 2- to 3-back tests of working memory would be present in this patient population. Therefore, we completed the investigation with functional neuroimaging and used a paradigm of systematically increasing the demand of the working memory test from 1-back to 3-back.

## Materials & methods

### Study participants

For inclusion, participants had to be aged 60–70 years with no self-reported cardiovascular disease or dementia based on registry data. The participants with type 2 diabetes were recruited from local diabetes registers. Participants on oral hypoglycaemic treatment were not excluded, but they had to refrain from taking the medication the morning prior to the investigation. Participants receiving insulin treatment were excluded. The controls were matched to this group by age and sex and randomly selected from a population with normal oral glucose tolerance test (OGTT) results in a local population-based health survey [25]. A total of 313 persons with type 2 diabetes and 273 controls received an invitation to participate by letter. Volunteers who met the inclusion criteria were invited by letter to the screening procedure described below. Participants were given both written and oral information about the study and provided written informed consent before the first screening visit. The Regional Ethical Review Board in Umeå, Sweden, approved the study.

### Screening

A questionnaire was distributed by mail that asked the participants about their height and weight, history of cardiovascular disease, whether they had participated in memory testing studies, prior head injuries, medications, problems with venous access, or if they had any diagnosis of dementia or diabetes. The participants also completed a depression-screening questionnaire (Centre for Epidemiologic Studies Depression Scale; CES-D) [26]. The exclusion criteria at this step of the selection procedure were high body weight (BMI >35 kg/m^2^), cardiovascular disease, dementia, a history of hospitalisation for head injury, earlier participation in cognitive studies, depression (CES-D score >15), medications that could influence cognitive function, and insulin treatment.

Sixty people with type 2 diabetes and 58 controls who were eligible for inclusion were invited to a screening visit. Venous blood samples were collected to measure blood haemoglobin and serum levels of thyroid stimulating hormone, thyroxine, cobalamin, folate, and creatinine to exclude conditions that could affect memory and individuals with severe renal failure. A resting electrocardiogram was obtained to exclude cardiac diseases, such as previous myocardial infarction. A questionnaire was administered regarding marital status; education level; current diseases; family history of cardiovascular disease, diabetes, and dementia; and lifestyle factors, such as smoking, snuff use, alcohol intake, and physical activity level as determined by the International Physical Activity Questionnaire. Physical activity was expressed as metabolic rate MET-min per week (level × minutes of activity × events per week). Education level was categorised as basic (completion of 9 years of compulsory education), medium (completion of 12 years of education), or high (graduation from university).

In the control group, a standardised OGTT was performed after overnight fasting to measure 2-hour plasma glucose after a 75-g glucose load to exclude individuals with undiagnosed diabetes. Plasma glucose was measured in capillary plasma using a Reflotron bench-top analyser (Boehringer Mannheim GmbH, Mannheim, Germany). HbA1c was measured in the group with type 2 diabetes (National Glycohemoglobin Standardization Program) as a percentage and mmol/mol (International Federation of Clinical Chemistry). All participants underwent a physical examination of the heart and lungs, and we measured their blood pressure, pulse rate, and waist circumference. A Mini Mental State Examination [27] was performed and participants with signs of impaired cognitive function (score <24) were excluded. All participants underwent *APOE* (gene map locus 19q13.2) genotyping using TaqMan Allelic Discrimination Technology. Genotypes were obtained for the two single nucleotide polymorphisms used to unambiguously define the ε2, ε3, and ε4 alleles (rs7412 and rs429358).

After the screening visit, 36 participants with type 2 diabetes (18 women and 18 men; mean age±SD 66.0±2.2 years) and 34 controls (17 women and 17 men; 65.8±0.8 years) were included in the first part of the study.

Sample size was calculated based on the results of a previous study [28]. The intention was to detect a between-group difference of 1.0 in the memory test z-score with a standard deviation of 1.4. The power was 0.80 at a 5% significance level. Therefore, we needed 31 participants in each group.

### Clamp methods

Memory function was tested under two metabolic conditions: a hyperglycaemic clamp [29,30] and a placebo clamp (saline infusion). For the hyperglycaemic clamp, a cannula was inserted into an antecubital vein for glucose infusion. To obtain arterialised venous blood samples, a cannula was inserted in the retrograde direction into the contralateral dorsal hand vein and maintained in a heated box at 50°C. Blood glucose concentrations were increased by a body surface area–adjusted intravenous bolus of 50% glucose solution over 1 minute, followed by continuous infusion of 20% glucose solution. Plasma glucose, insulin, and C-peptide concentrations were measured continuously (S1 Fig). The target was to reach a stable concentration of 15 ± 0.5 mmol/l of blood glucose in two subsequent measurements. Arterialised capillary blood samples were drawn for glucose and insulin analyses (HemoCue Glucose 201 RT). The experimental setting for the placebo clamp was the same as for the hyperglycaemic clamp, but the infusion consisted of a physiological sodium chloride solution instead of glucose (S2 Fig). All clamps were performed in a randomised order at least 2 weeks apart. All participants fasted overnight and abstained from exercise for 8 hours before the clamp sessions.

Memory assessments lasted approximately 20 minutes and were performed by a nurse who was blinded to the type of clamp and the glycaemic status of the participant. Testing began approximately 70 (range 60–95) minutes into the clamps when a stable blood glucose concentration was achieved. The memory tests were finished after approximately 90 (range 70–120) minutes.

### Memory testing

Episodic, semantic, and working memory were assessed as described previously [31]. The assessment of episodic memory included sentence and word recall tests. In the sentence learning assessment, the participants were instructed to learn a list of 16 short sentences expressed as commands (e.g., “Lift the book”), followed by a free recall test 2 minutes later. The number of correctly recalled verbs and nouns was recorded. To test the free recall of words, a list of 12 words (e.g., brother, game, letter) were read one at a time every other second and the participants instructed to try to remember the words. After a short pause (45 seconds), the subjects were asked every other second to recall one word at a time. The assessment of semantic memory included three tests of word fluency. Over 1 minute, the participants were told to generate as many words as possible according to the following instructions: (a) words with A as the initial letter, (b) five-letter words beginning with M, and (c) professions starting with the letter B.

In the behavioural part of the study, working memory was assessed by a single 2-back test. In the 2-back test, a list of 40 words was read to the participant. After each of the words, the participant had to answer yes or no as to whether it was the same as the word presented two words earlier. The content of the memory tests differed for the two clamp conditions to avoid a learning effect.

### Statistical analysis

Descriptive data are presented as means with standard deviations (SDs). Differences in means and proportions between the group with type 2 diabetes and control group were tested for continuous and categorical variables using the Student’s *t*-test and *χ*^2^ test, respectively. *P* < 0.05 was considered significant; no allowance was made for multiplicity of tests.

We standardised the results of the individual episodic and semantic memory test scores by creating a composite z-score: score minus the mean score of the control group during the placebo clamp, divided by the SD of the control group. Memory scores were determined by adding each z-transformed memory score and dividing the result by the number of included tests. However, working memory was measured with one 2-back test and the z-transformation was redundant.

A mixed-models method was used to calculate repeated measurements of memory during the two clamp conditions. We used a basic model including measurement number (time 1/time 2) to adjust for the learning effect between the first and second clamp. We also included group (diabetes/control) to examine differences between groups and clamp (placebo clamp/hyperglycaemic clamp) and evaluate the clamp effect for each of the three different memories. In the univariate analysis, we used this basic model and then added the potential confounding factors. We used IBM SPSS Statistics 25 for all statistical calculations in the first part of the study.

### Screening for fMRI

All eligible participants (n = 70) from the first part of the study were invited to participate in the fMRI study. Therefore, no further power calculation was made. Twenty-seven participants agreed to participate, 22 of whom (10 with type 2 diabetes and 12 controls) completed both clamp sessions.

### Clamp methods for fMRI

During the clamps, also in the MRI camera, C-peptide and plasma glucose concentrations were measured in samples collected at about every 10 minutes. The clamps lasted for about 240 minutes.

### Memory testing in the fMRI part of the study

During fMRI, working memory was tested under the two metabolic conditions described above (i.e., hyperglycaemic clamp and placebo clamp). When the blood glucose concentration stabilised, approximately 130 minutes into the clamp, participants entered the MRI camera and memory assessments were performed. Memory testing and the fMRI sequence was completed at approximately 180 minutes.

Participants received instructions on the n-back task before entering the fMRI scanner. In contrast to the previous assessment of verbal memory, we used fMRI tasks that were used in previous studies [31,32] and an imaging protocol from past fMRI studies [22,33]. The participants viewed stimuli on a screen via a mirror mounted on a head coil. The stimuli were presented in white on a black background using E-prime 2.0.10 software (Psychology Software Tools, PA, USA), which also recorded behavioural performance. The n-back task consisted of 21 lists (seven each of the 1-back, 2-back, and 3-back lists) in random order [33]. The lists consisted of 10 items (digits 1–9), and each item was presented for 1.5 s, with a cross presented for 0.5 s between each item. A rest period of 20 s occurred three times, during which a cross was presented. Participants indicated whether each item in the list matched an item that occurred one, two, or three items back. Participants used their right hand to respond, with the index finger indicating “yes” and middle finger indicating “no”. The maximum number of correct responses was 168.

### MRI data acquisition

Images were acquired using a 3T Discovery 750 scanner from General Electric. Functional T2*-weighted images were obtained as a single-shot gradient echoplanar imaging sequence used for BOLD contrast imaging (repetition time: 2000 ms, acquiring 37 slices; echo time: 30 ms; flip angle: 80°; field of view: 25 × 25 cm; 96 × 96 voxels zero-filled to 128 × 128 voxels; and 3.9-mm slice thickness). Ten dummy scans were automatically collected and discarded prior to image acquisition to eliminate signals arising from progressive saturation. Anatomical T1-weighted images were obtained with a Fast SPGR sequence (repetition time: 8.2 ms; echo time: 3.2 ms; 25 × 25 cm field of view; 512 × 512 voxels; 1-mm slice thickness). After acquisition, the DICOM images were converted to NIfTI format.

### Statistical analysis of the fMRI results

Working memory in the neuroimaging study was calculated as the number of correct answers in relation to the maximum number of possible correct answers. We evaluated differences between groups using Fisher’s exact tests and within groups with one-sided t-tests.

C-peptide and plasma glucose concentrations were linearly interpolated to 1-minute resolution and an area under curve (AUC) over the time for the fMRI-sessions were calculated. Differences between hyperglycaemia and placebo AUCs were used in two-sided t-tests.

The MRI data were pre-processed and analysed in SPM12 with assistance from the in-house program DataZ. Both SPM12 (http://www.fil.ion.ucl.ac.uk/spm; Wellcome Department of Imaging Neuroscience, London, UK) and DataZ ran under MATLAB R2014b (MathWorks, Inc., Natick, MA, USA). The files from the first scan were segmented into grey and white matter likelihood maps in Dartel import space. From these files, we created a Dartel template for the group of participants and flow-field file for each participant [34]. The flow-field file is a voxel-to-voxel transformation from native space to Dartel-template space. The fMRI files were slice time-corrected to the first slice in each volume, and then movement-corrected with SPM realign and unwarp functions. The fMRI files were then co-registered to the MRI file from the first scan. We applied the flow-field transformation and an affine transformation to the fMRI files, normalising the files to the MNI-space with 2-mm resolution. Finally, we spatially smoothed the fMRI files by convolving them with an 8-mm Gaussian filter.

To analyse the fMRI data for each participant, a general linear model was set up with a block regressor for each of the three conditions and six nuisance regressors. The block regressors were convolved with the canonical haemodynamic response function. The nuisance regressors consisted of the parameters from the movement correction (three translation variables and three rotation variables). The general linear model generates a beta-value for each voxel and regressor, reflecting the impact of the regressor on the BOLD signal in that voxel. The differences in beta values for a 3-back-regressor and 1-back-regressor were used in a group analysis of all participants (both controls and individuals with type 2 diabetes) in the placebo session, utilizing t-test. The AUC C-peptide levels was used as covariate. We set a voxel threshold of p < 0.001 (uncorrected for multiple corrections) and excluded clusters with an extent of fewer than 10 voxels. We plotted responses for each group and condition in two peaks in regions identified in the group analysis as having a higher BOLD signal during 3-back than 1-back. Based on the values of the two peaks of interest, two ANOVAs was set up with one group factor (control vs. type 2 diabetes) and one condition factor with six items (1-back, 2-back, and 3-back during placebo and 1-back, 2-back, and 3-back during hyperglycaemic clamp). A control analysis with age and sex as additional covariates in the fMRI-model during placebo was performed.

## Results

As expected, participants with type 2 diabetes had significantly higher BMI, larger waist circumference, higher fasting blood glucose, were less physically active, and more often had a family history of diabetes compared to controls (Table 1). Notably, the participants with type 2 diabetes had good metabolic control and well-controlled blood pressure. In terms of risk factors for dementia, we found no difference in education level, family history of dementia, or proportion of *APOE* ε4 between the two groups (Table 1).

**Table 1:** Characteristics of the study population.

	Type 2 DM Group (n = 36)	Control Group (n = 34)	P-value
Age, years	66.0 (2.20)	65.8 (0.82)	NS
Sex, women	18(50%)	17(50%)	
High education level	7 (19%)	6 (18%)	NS
Married	28 (78%)	30 (88%)	NS
Diabetes duration, years	7.09 (4.17)	–	–
BMI, kg/m^2^	27.9 (3.51)	26.2 (2.97)	0.03
Waist circumference, cm	101 (11)	91 (10)	<0.001
Family history of diabetes	18 (50%)	8 (24%)	0.013
Family history of cardiovascular disease	12 (33%)	11 (32%)	NS
Family history of dementia	6 (17%)	10 (29.4%)	NS
Current smoker	3 (8%)	5 (15%)	NS
*APOE* ε4 homozygotes	1 (3%)	1 (3%)	NS
*APOE* ε4 heterozygotes	10 (29%)	8 (24%)	NS
Alcohol consumption			NS
Never	6 (17%)	5 (15%)	
≤1 drink per month	15 (42%)	8 (24%)	
2–4 drinks per month	12 (33%)	10 (29%)	
2–3 drinks per week	3 (8%)	11 (32%)	
Physical activity, MET min/week	1736 (1479)	2966 (3093)	0.041
Systolic blood pressure, mmHg	137.4 (14.2)	136.2 (16.3)	NS
Diastolic blood pressure, mmHg	79.5 (8.0)	82.0 (9.5)	NS
MAP, mmHg	98.8 (8.0)	100.0 (10.9)	NS
Fasting plasma glucose, mmol/l	7.49 (1.46)	5.00 (0.63)	<0.001
2-hour plasma glucose, mmol/l	–	5.02 (1.37)	–
HbA1c, mmol/mol	52 (8.8)	37 (2.8)	<0.001
HbA1c, %	6.0 (0.8)	4.6 (0.3)	<0.001
HOMA-IR	0.76 (0.57)	0.28 (0.15)	<0.001
Delta glucose, glucose clamp, mmHg	7.88 (1.40)	9.71 (1.58)	<0.001
AUC glucose, placebo clamp	892 (151)	626 (62)	<0.001
AUC glucose, glucose clamp	1841 (48)	1762 (89)	<0.001
AUC insulin, glucose clamp	3940 (2972)	6035 (3194)	0.006
AUC C-peptide, glucose clamp	218 (95)	313 (88)	<0.001
Medical treatment of type 2 diabetes			
Metformin	25 (69%)		
Other oral antidiabetic	4 (11%)		
Diet	7 (19%)		

Data are reported as n (%) or mean (SD). NS: Not significant; DM: Diabetes mellitus; *APOE* ε4: Apolipoprotein E ε4 allele; MET: Metabolic rate; MAP: Mean arterial pressure; HOMA-IR: Homeostatic Model Assessment of Insulin Resistance; Delta glucose: Mean of three glucose measurements during memory testing minus three fasting glucose measurements before glucose infusion; AUC: Area under the curve from time 0 to time 120.

The dynamic changes in blood glucose, insulin, and C-peptide during the hyperglycaemic clamp are presented by group in S3 Fig.

Across conditions, the control group had better working memory (mean 2-back score, 33.15 ± 0.45) than the participants with type 2 diabetes (mean 2-back score, 31.8 ± 0.44, adjusted mean difference B = 1.31, F (df^1^1 df^2^68) = 4.31; p = 0.042). Physical activity was included in the basic model, which attenuated the association, but the difference between the groups was still significant (p = 0.049). Including the area under the curve (AUC) for insulin and C-peptide in the model did not alter the results.

Episodic and semantic memory did not differ between groups during either of the clamp conditions (Table 2). Thus, acute hyperglycaemia was not associated with a significant change in working, episodic, or semantic memory in either group.

**Table 2:** Memory differences between groups by clamp.

	Type 2 DM Group (n = 36)	Control Group (n = 34)	P-value
**Placebo clamp**			
Episodic memory, z-score	–0.13 (0.8)	–0.00 (0.8)	NS
Semantic memory, z-score	–0.14 (0.9)	0.00 (0.6)	NS
Working memory, 2-back score	31.78 (3.6)	33.47 (2.6)	0.028
**Hyperglycaemic clamp**			
Episodic memory, z-score	–0.13 (0.8)	–0.03 (0.8)	NS
Semantic memory, z-score	–0.09 (0.9)	0.10 (0.9)	NS
Working memory, 2-back score	31.89 (2.8)	32.82 (3.0)	NS

Data are reported as mean (SD). NS: Not significant; DM: Diabetes mellitus.

### Neuroimaging

The dynamic changes in plasma glucose, and C-peptide during the hyperglycaemic and placebo clamps are presented by group and treatment in S4 Fig.

**Fig 1:**
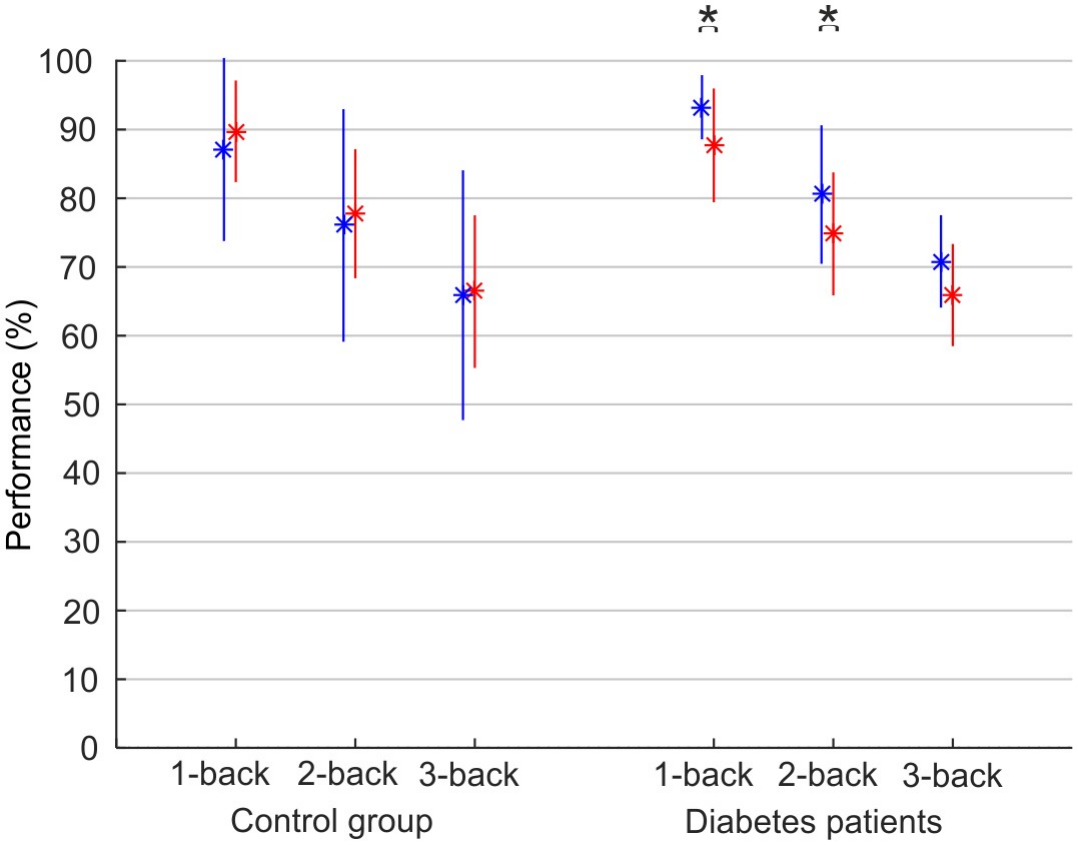
Working memory performance in the fMRI investigation during hyperglycaemic or placebo clamp. Performance was measured as the percentage of correct answers during n-back tests (1-back, 2-back, and 3-back). The placebo was saline infusion (blue); hyperglycaemic clamp is shown in red. Data are reported as mean±SD. *p<0.05 between saline infusion and hyperglycaemic clamp.

In the fMRI study, we evaluated the impact of hyperglycaemia on working memory performance. The characteristics of the population are presented in Table 3. Working memory was significantly reduced in the type 2 diabetes group during hyperglycaemia compared to the placebo clamp when assessed by 1-back and 2-back (p = 0.029 and p = 0.023, respectively), and a similar trend was found for 3-back (p = 0.051; Fig 1). We found no difference in working memory between the two clamps in the control group.

**Table 3:** Characteristics of the population assessed by neuroimaging.

	Type 2 DM Group (n = 10)	Control Group (n = 12)	P-value
Women, n	6 (60%)	8 (60%)	
Age, years	69 (1.9)	69 (0.7)	NS
BMI, kg/m^2^	25.8 (4.0)	25.7 (3.5)	NS
Systolic blood pressure, mmHg	136.2 (11.6)	138.2 (14.7)	NS
Diastolic blood pressure, mmHg	75.4 (4.6)	78.7 (9.8)	NS
HbA1c	51.2 (8.2)	40 (6.0)	0.001
P-glucose, placebo clamp, mmol/l	6.9 (1.8)	5.0 (0.7)	0.009
P-glucose, glucose clamp, mmol/l	15.19 (1.4)	13.0 (2.8)	0.027
Delta P-glucose, glucose clamp, mmol/l	7.5 (2.4)	6.2 (1.5)	NS
Glucose, ΔAUC (glucose-placebo clamp), min*mmol/l	164.65 (42.04)	136.31 (23.42)	0.060
C-peptide, ΔAUC (glucose-placebo clamp), min*nmol/l	27.83 (16.98)	54.98 (23.37)	0.0062

Data are reported as n (%) or mean (SD). DM: Diabetes mellitus; NS: Not significant; P-glucose, plasma glucose; Delta P-glucose: Difference between mean plasma glucose in the clamp during memory testing and fasting plasma glucose. Initial working memory represents the 2-back scores in the first part of the study.

**Fig 2:**
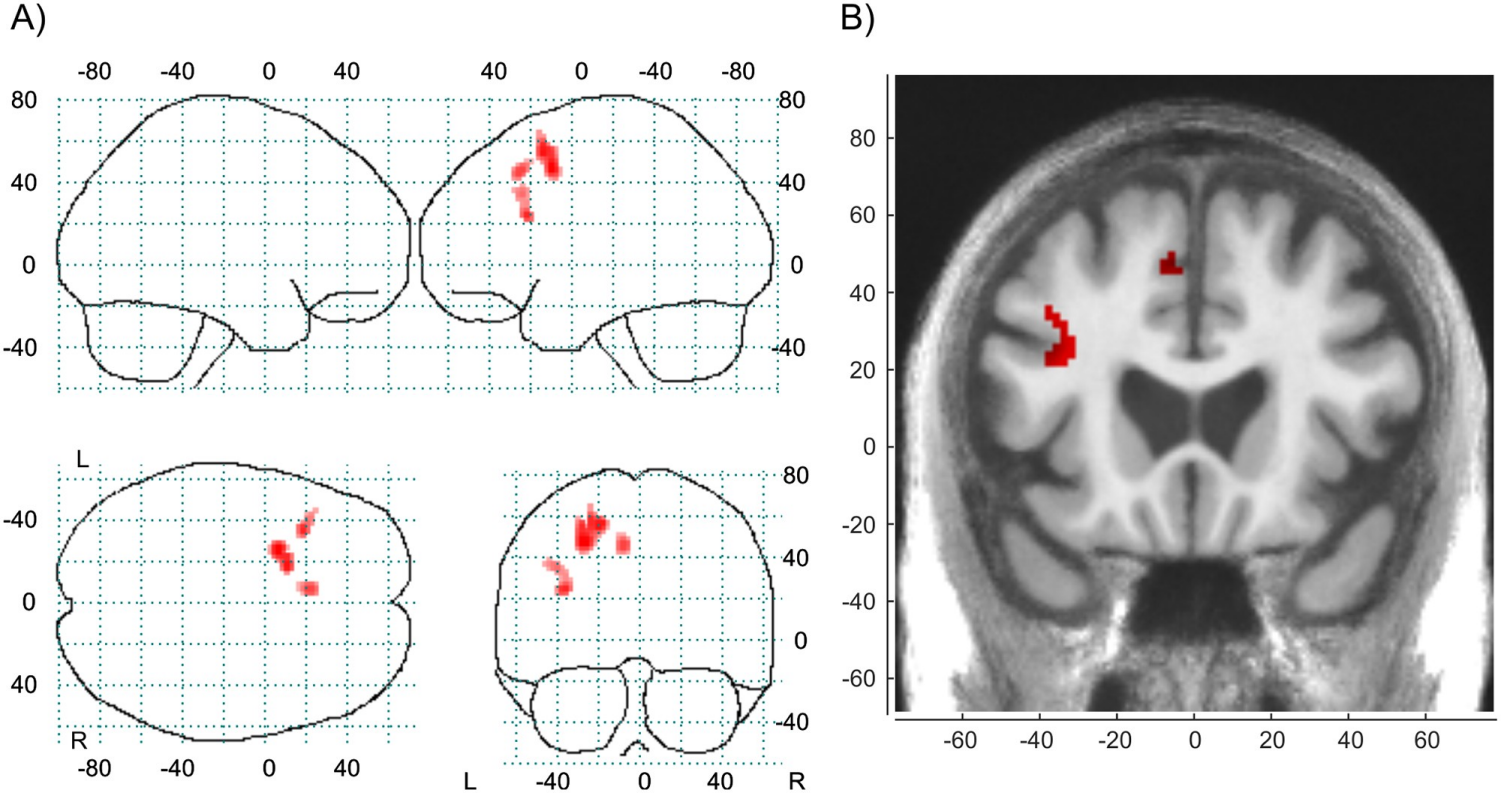
Load-dependent working memory network. Working memory load effect. 3-back show stronger BOLD-response than 1-back at placebo with a voxel threshold at p (uncorrected) < 0.001, cluster size ≥ 10 voxels. (A) Glass brain. (B) Slice image at y = 20.

The first step in the fMRI analyses was to find areas in which the BOLD-response increased with higher working memory load under normal conditions, i.e., during the placebo clamp. We found that the BOLD signal in the left DLPFC and dorsal ACC/pre-SMA increased as a function of working memory load as depicted in Fig 2. A detailed description of the cluster information is given in Table 4. The control analyses with AUC c-peptide, age and sex as covariates in the model gave the same clusters as with only AUC c-peptide as covariate in the model. The left DLPFC peak had a reduced statistics from T = 4.10 to T = 4.09. The dorsal ACC/pre-SMA peak had a reduced statistics from T = 3.99 to T = 3.89. We concluded that age and sex as covariate was not necessary.

**Table 4:** Detailed description of the cluster information.

Cluster				Peak voxel in cluster			
Cluster no	Size (voxels)	Neuromorphometrics label in SPM	Part of cluster	T-value	x	y	z
1	142	L. Superior frontal	37%	4.24	-26	8	46
		L. Middle frontal	32%				
		L. White matter	31%				
2	46	L. White matter	63%	4.10	-34	20	24
		L. Middle frontal	28%				
		L. Opercular part of the inferior frontal	9%				
3	35	L. Supplementary motor (dorsal ACC/pre-SMA)	63%	3.99	-6	22	46
		L. White matter	23%				
		L. Superior frontal	14%				

Clusters (aggregated voxels) visualised in Fig 2 with information about regions within clusters and their size share (http://neuromorphometrics.com).

**Fig 3:**
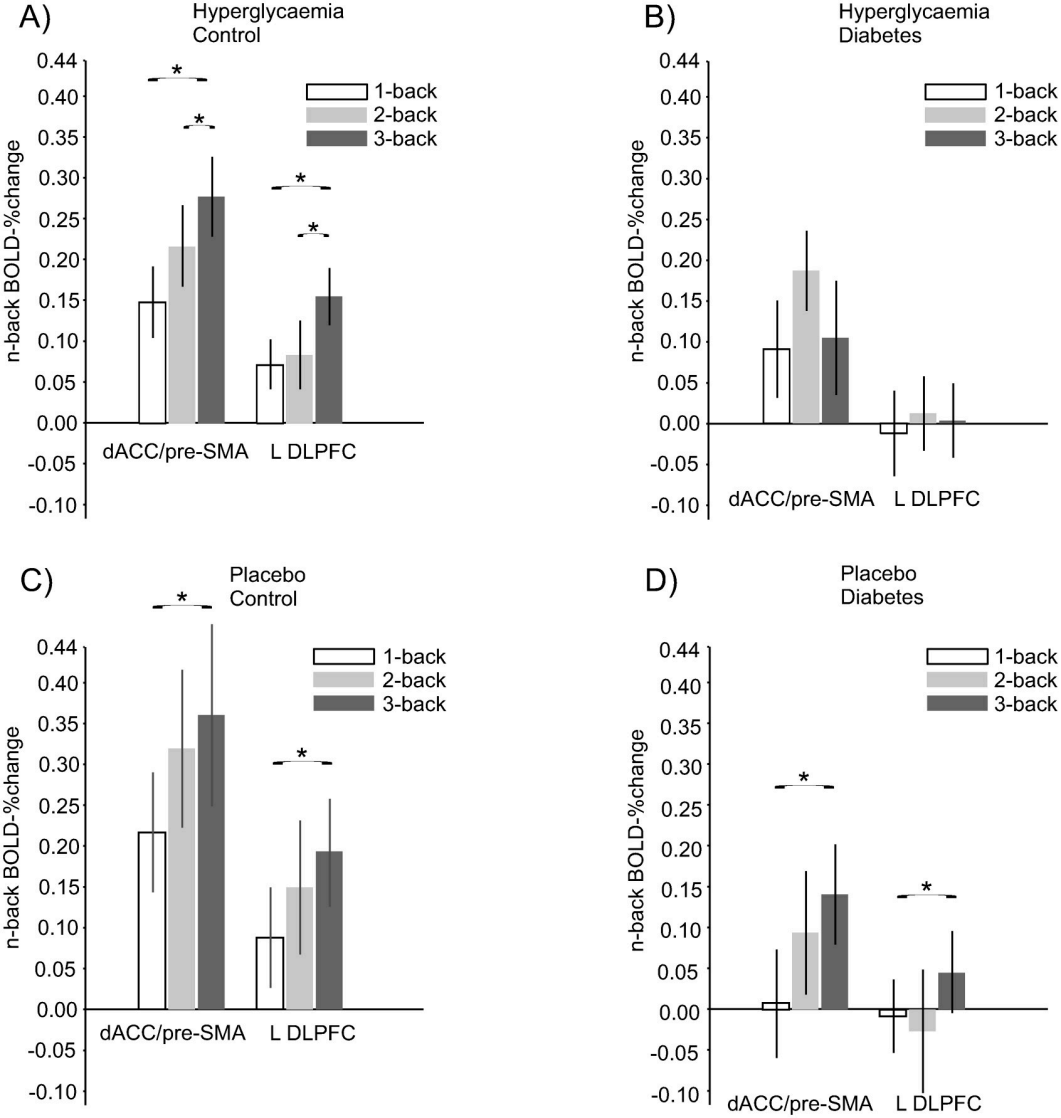
Load-dependent PFC BOLD functions during a test of working memory. (A, B) N-1/2/3-back BOLD signal relative to baseline in controls and participants with type 2 diabetes during hyperglycaemia (C, D) an placebo clamp. Data are presented as mean ± SE. dACC = dorsal anterior cingulate cortex; pre-SMA = pre-supplementary motor area; L = Left; DLPFC = dorsolateral prefrontal cortex.

Next, we examined the load functions in the two frontal peaks for controls and participants with type 2 diabetes during the placebo and hyperglycaemia clamp. The controls had a numeric load response (3 > 2 > 1) during both conditions, whereas the type 2 diabetes group had a similar response during placebo but with a decrease in the frontal BOLD response from the 2- to 3-back load during hyperglycaemia (Fig 3). In an ANOVA of the BOLD-response in the peaks, the main effect of groups showed a difference in the left DLPFC peak (F(1,20) = 4.53, p = 0.046) and a trend in the dorsal ACC/pre-SMA peak (F(1,20) = 4.16, p = 0.055).

## Discussion

We studied the acute effect of hyperglycaemia on working, episodic, and semantic memory, and the effect of hyperglycaemia on brain activity during working memory. First, we found that, across both clamp conditions, the control group had better working memory than the group with type 2 diabetes. Second, in the fMRI study, we found that participants with type 2 diabetes had a significantly reduced performance during hyperglycaemia and a decreased load response to the most demanding test during the hyperglycaemic clamp. This finding is in line with a previous study showing marked differences in brain activation patterns when healthy adults were tested using a spatial working memory task with three load levels [17]. Taken together, our findings suggest that individuals with type 2 diabetes may have altered glycaemia-induced brain activity in specific areas of the PFC. Importantly, higher cognitive loads were necessary to reveal the failure to upregulate activity in the PFC in response to higher working-memory demands under hyperglycaemia in people with type 2 diabetes.

Only a few studies have measured cognitive function using a standardised hyperglycaemic clamp (hyperinsulinaemic euglycaemic clamp) technique [13,14,16]. None of the previous studies used a standardised placebo clamp as a reference for memory function or had a control group. Our results from the first part of the study are in line with those of Draelos et al. [13] and Pais et al. [14], who found no differences in memory performance during hyperglycaemia. In contrast, Sommerfield et al. [16] reported impaired working memory and attention with hyperglycaemia in line with our fMRI study during the most demanding working memory tests. These earlier studies had considerable methodological differences, which may influence the interpretation of the results.

Sommerfield et al. [16] reported on a study population with younger participants and used other working memory tests. Furthermore, they and others [13,14,16] used a hyperinsulinaemic euglycaemic clamp to achieve euglycaemia. This clamp is designed to evaluate insulin secretion and resistance by infusing insulin [29]. Thus, the observed effect on cognitive function could be a result of hyperinsulinaemia in the euglycaemic state because insulin itself could affect cognitive function [35]. Notably, He et al. [21] found that 12 participants with type 2 diabetes performed the n-back task as well as matched controls during hyperglycaemia. These authors also reported a significant load-by-group interaction of brain activation in the right dorsolateral PFC, left middle/inferior frontal gyrus, and left parietal cortex. Participants with type 2 diabetes exhibited hyperactivation in the 2-back, but not the 0-back, which in similar to our findings during the placebo clamp.

We used a clamp technique that allowed us to control glucose concentrations throughout the experiment, whereas He et al. used a single plasma glucose measurement taken before the scan as a proxy for the glucose concentration during the test [21]. Notably, our study participants were older, with a longer disease duration than the population in He et al.’s study, in which the patients with type 2 diabetes were newly diagnosed.

Our fMRI results are in line with the study by Huang et al. [18] who found a similar load pattern among people with type 2 diabetes as in our study where DLPFC was affected. However, the changes were mainly localized to the right hemisphere which might be explained by their test of the spatial working memory while we used the n-back test. Zanchi et al. [19] also used the n-back test and found a decreased activation in ACC, similar to our findings, after ingestion of glucose but not fructose which they explained by the differences in metabolism of glucose and fructose [19]. They found no effect of either glucose or fructose on behavioral tasks, but that could be due to the very small sample size. Chen et al. [36], who found that more regions of diminished activation in the frontal cortex were observed with increasing task difficulty in patients with type 2 diabetes compared to controls when working memory was tested. The authors speculated that the observation could be due to impairments in neuronal density or viability, glial proliferation, and demyelination in diabetes [36]. The investigation most comparable to our fMRI study is a very recent study by Omladic et al. [20], who studied the impact of acute hyperglycaemia using a similar clamp protocol as in our study; assessed working memory during normal blood glucose and during hyperglycaemia induced by a hyperglycaemic clamp. Their population was 20 adolescents (mean age 15 years) with type 1 diabetes and 20 healthy controls whose spatial working memory was tested and brain activity assessed by fMRI. Hyperglycaemia reduced the spatial working memory in participants with type 1 diabetes, whereas the control group had improved working memory during hyperglycaemia. In addition, brain activity significantly decreased in the type 1 diabetes group but slightly increased in the controls; a pattern that was very similar to our results [20].

Finding that only working memory was affected by acute hyperglycaemia in patients with type 2 diabetes underscores our previous observations [7,9] that some brain areas could be more sensitive to glucose perturbation in certain study populations. These findings may also be influenced by age; Awad et al. [37] exposed 21-year old undergraduate students (n = 74) to hyperglycaemia, which resulted in a poorer result on all memory tests and, most significantly, on the most demanding tasks, which is similar to our observation in the fMRI study.

When added to previous results [20,21,36], our findings suggest a neuronal activity threshold that is affected by hyperglycaemia in individuals with type 2 diabetes. A key player in upholding neuronal activity in metabolically active brain regions is glucose transporter 3 (GLUT-3) [38]. GLUT-3 is downregulated in the brain in type 2 diabetes [39], and patients with type 2 diabetes could be hypothesised to have an insufficient capacity to increase their neuronal activity due to insufficient GLUT-3 function in specific brain regions during more challenging memory tasks. The difference in working memory challenge may also explain why hyperglycaemia did not affect 2-back in the behavioural part of the study but did so among patients with type 2 diabetes in the imaging study, in which brain activity decreased in the 3-back test.

Because BOLD signals depends on blood flow, it could be argued that our results reflect an effect of changes in regional blood flow in the brain [40]. We think this is unlikely because of a clear dose-response pattern in the BOLD signal for the type 2 diabetes group during the placebo clamp. Moreover, an acute elevation of plasma glucose would be unlikely to have an immediate impact on blood flow in a specific brain region, as we detected no differences in activated areas between the placebo and hyperglycaemic clamps.

A major strength of our study is the well-defined population without any concomitant diseases that could impair memory function. In addition, this study is the first to use a placebo clamp in this experimental setting to control for the stressful situation. Furthermore, we challenged participant working memory using 3-back in the fMRI study, which constitutes a higher cognitive load than previous studies. However, this study also has limitations. First, the selection process for the type 2 diabetes group led to a metabolically well-controlled group, potentially reducing external validity. In addition, we chose to assess hyperglycaemia with a hyperglycaemic clamp as in previous studies, but the endogenous insulin production was not supressed as in a hyperinsulinaemic euglycaemic clamp. Instead, insulin was measured in both parts of the study and was adjusted for in the statistical analyses. Furthermore, we did not use an extensive battery of episodic memory tests, such as tests of recognition memory, or administer any IQ test. Moreover, the sample size in the fMRI study was limited, which could result in a lack of power. We also acknowledge that the different versions of n-back tasks prevented strong comparisons of the behavioural patterns in the main study and the fMRI study. Finally, the comparisons in Tables 1 and 3 were not adjusted for multiple comparisons, which could increase the risk of type 1 errors.

## Conclusion

In our study, acute elevation of glucose concentrations affected neuronal and behavioural function in participants with type 2 diabetes when their working memory was challenged. This finding implies that working memory and the ability to perform complex activities during hyperglycaemic episodes can affect everyday life. We can only speculate about how or if this deficit affects the everyday life of people with type 2 diabetes. What we do know is that individuals with diabetes can have difficulties dealing with more complex everyday activities, including keeping track of medications [41], which includes the activation of working memory. Whether hyperglycaemia accelerates these difficulties is yet to be determined. Therefore, large epidemiological studies assessing the effect of blood glucose variability on working memory in type 2 diabetes are warranted. To increase our understanding of the mechanisms underlying the association between glucoregulation and memory, more mechanistic studies, including positron emission tomography (PET) with metabolic ligands or fMRI spectroscopy during acute hyperglycaemia, are warranted.

## Supporting information

S1 Fig Design of the hyperglycaemic clamp. BP: Blood pressure, ECG: Electrocardiogram, Pvc: Peripheral vein catheter.
(TIF)

S2 Fig Design of the placebo clamp. BP: Blood pressure, ECG: Electrocardiogram, Pvc: Peripheral vein catheter.
(TIF)

S3 Fig Dynamic changes in blood glucose, insulin, and c-peptide during clamping by diabetes status. (TIF)

S4 Fig Dynamic changes in blood glucose (A) and c-peptide (B) by diabetes status and clamp during neuroimaging. Blue staples are the intervals for the fMRI-sessions.
(TIF)
