# Supplementary figures and images for: Acute hyperglycaemia leads to altered frontal lobe brain activity and reduced working memory in type 2 diabetes
Source: PLoS One. 2021 Mar 19;16(3):e0247753. doi: 10.1371/journal.pone.0247753 (PMC7978337; doi:10.1371/journal.pone.0247753)

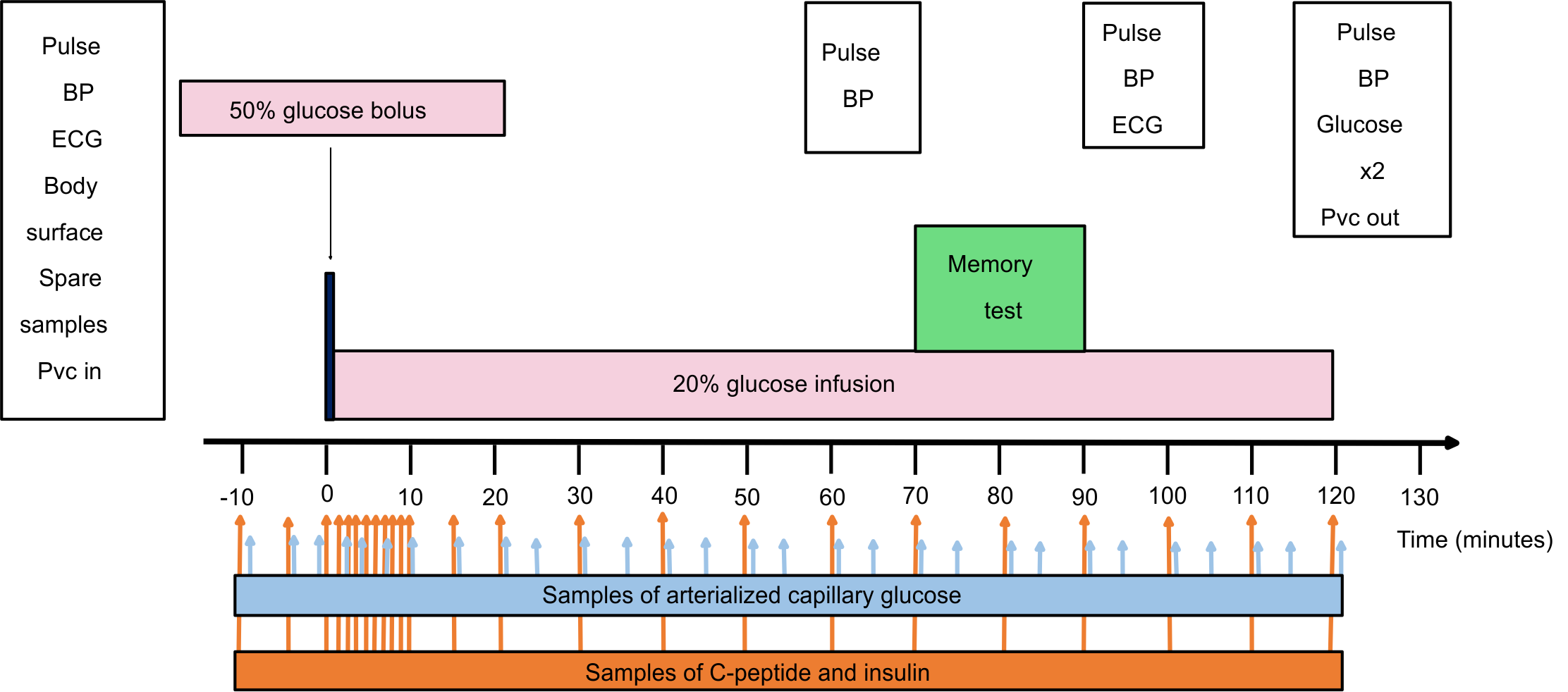

Supplement: S1 Fig — BP: Blood pressure, ECG: Electrocardiogram, Pvc: Peripheral vein catheter. (TIF) [file pone.0247753.s001.tif]

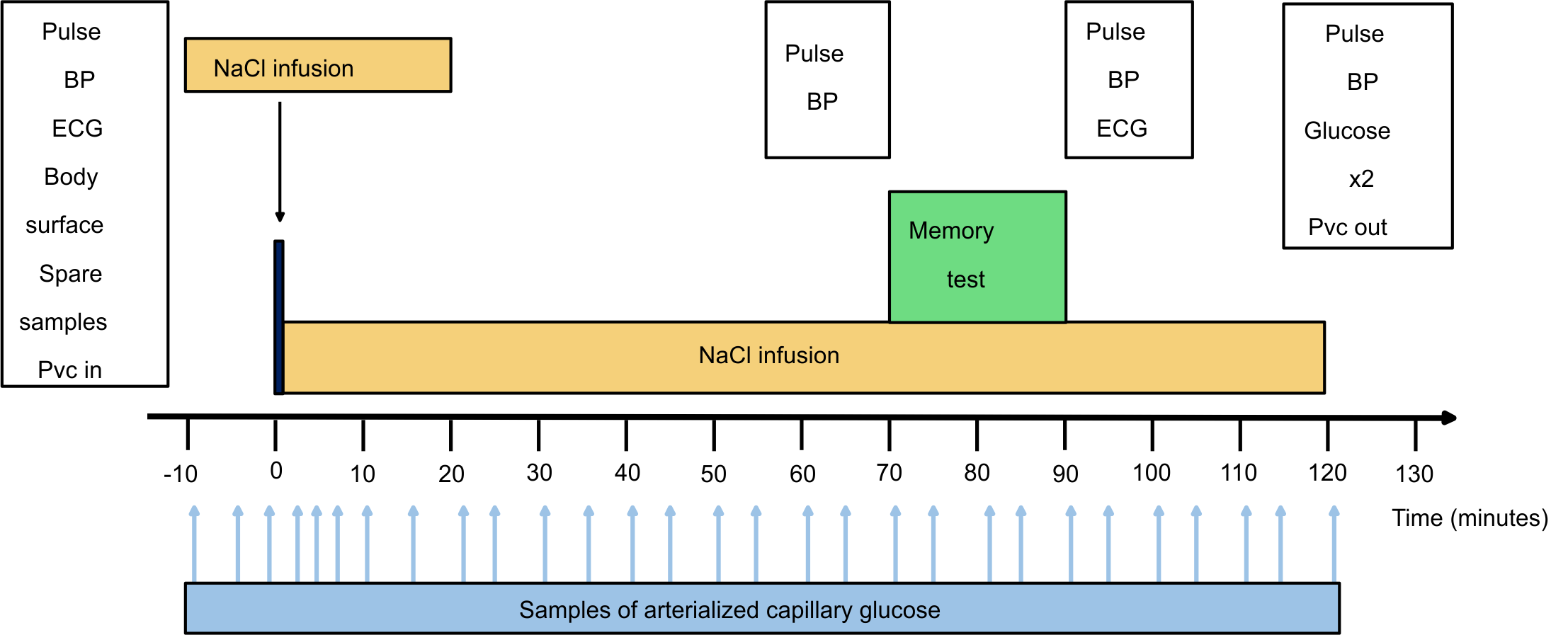

Supplement: S2 Fig — BP: Blood pressure, ECG: Electrocardiogram, Pvc: Peripheral vein catheter. (TIF) [file pone.0247753.s002.tif]

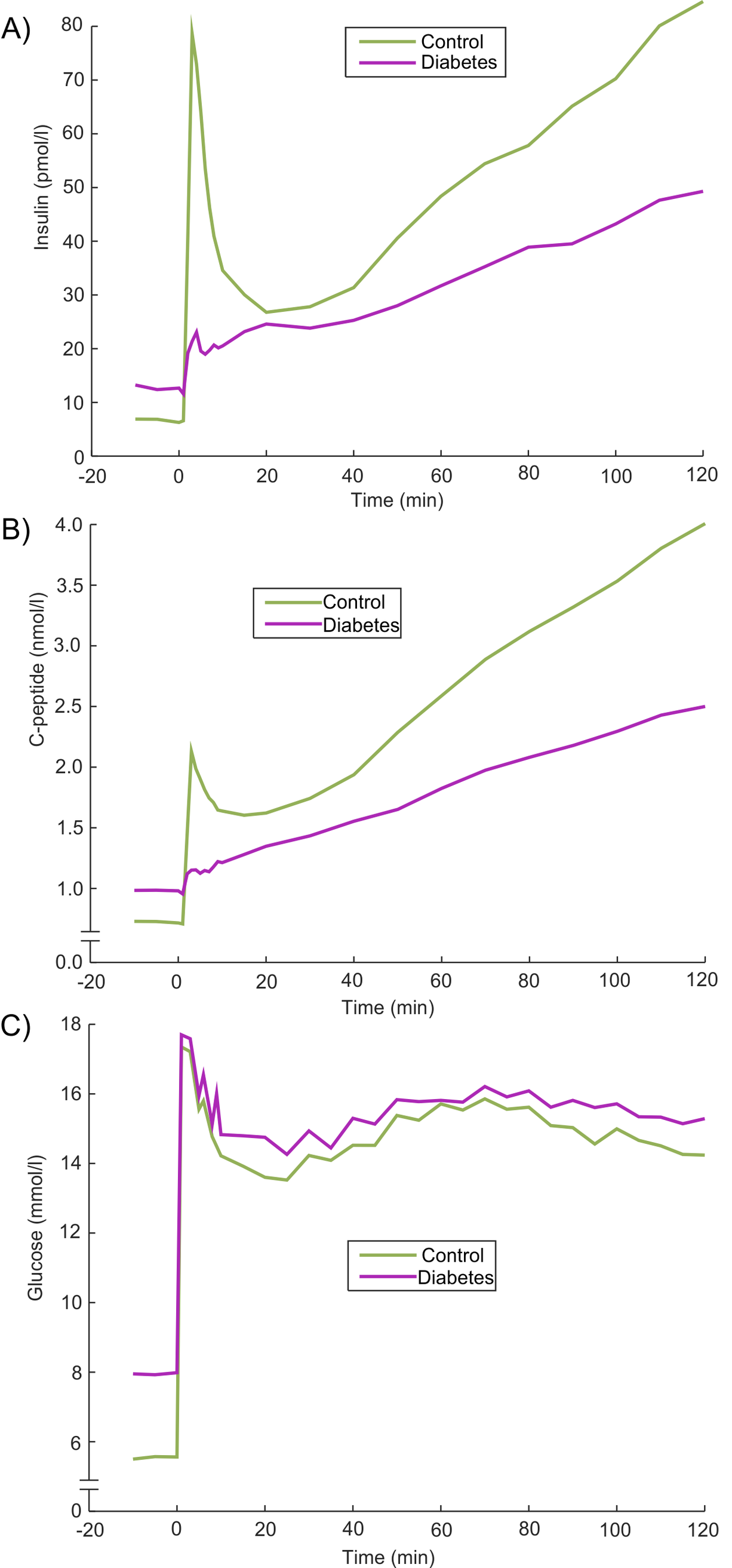

Supplement: S3 Fig — (TIF) [file pone.0247753.s003.tif]

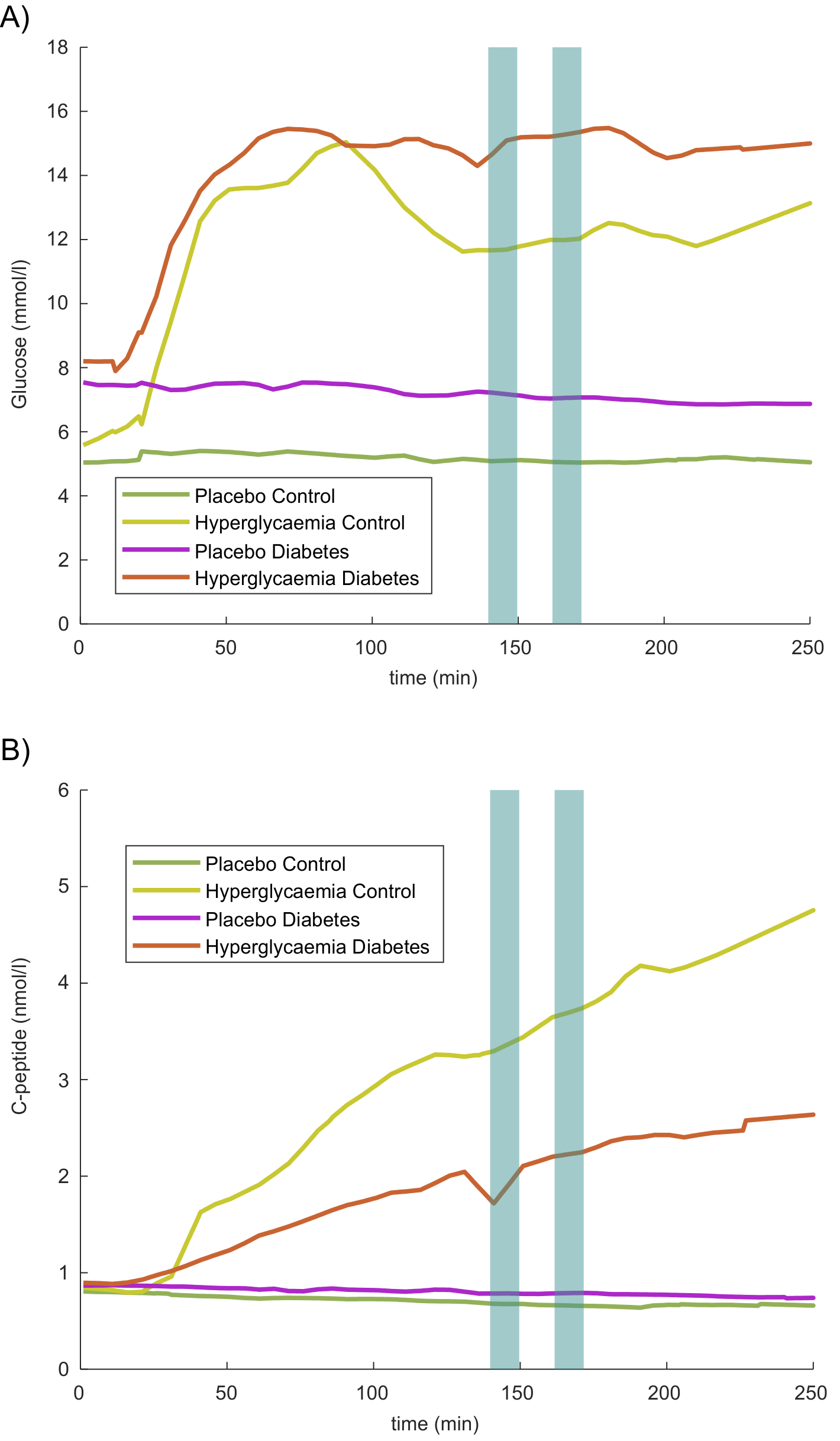

Supplement: S4 Fig — Blue staples are the intervals for the fMRI-sessions. (TIF) [file pone.0247753.s004.tif]
